# Quantum Information Theory on Sparse Wave Functions and Applications for Quantum Chemistry

**DOI:** 10.1021/acs.jpca.5c02137

**Published:** 2025-09-09

**Authors:** Davide Materia, Leonardo Ratini, Leonardo Guidoni

**Affiliations:** † Dipartimento di Scienze Fisiche e Chimiche, 201850 Università degli Studi dell’Aquila, Coppito, L’Aquila 67100, Italy; ‡ Dipartimento di Ingegneria e Scienze dell’Informazione e Matematica, Università degli Studi dell’Aquila, Coppito, L’Aquila 67100, Italy; § Dipartimento di Scienze Matematiche, Fisiche e Informatiche, Università degli Studi di Parma, Parma 43100, Italy

## Abstract

In recent years Quantum Computing prominently entered in the field of Computational Chemistry, importing and transforming computational methods and ideas originally developed within other disciplines, such as Physics, Mathematics and Computer Science into algorithms able to estimate quantum properties of atoms and molecules on present and future quantum devices. An important role in this contamination process is attributed to Quantum Information techniques, having the 2-fold role of contributing to the analysis of electron correlation and entanglements and guiding the construction of wave function variational ansatzes for the Variational Quantum Eigensolver technique. This paper introduces the tool SparQ (Sparse Quantum state analysis), designed to efficiently compute fundamental quantum information theory observables on post-Hartree–Fock wave functions sparse in their definition space. The core methodology involves mapping Fermionic wave functions to qubit space using Fermionic-to-qubits transformations and leveraging the sparse nature of these wave functions to evaluate observables and properties of the wave function. The effectiveness of SparQ is validated by analyzing the mutual information matrices of wave functions for the water molecule and the entropy of ∼10^2^ qubits describing the benzene molecule. This highlights its capability to handle large-scale quantum systems, limited mainly by the capabilities of quantum chemical methods used to retrieve the wave functions. The results indicate that quantum information theoretical analysis, so far limited to traditional tensor network methods and study of transition operators, can be applied to all post-Hartree–Fock wave functions, extending their applications to larger and more complex chemical systems.

## Graphical abstract

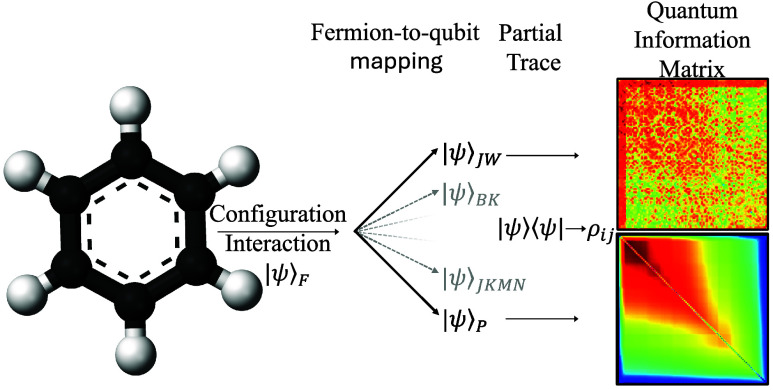

## 1 Introduction

In recent years, the rapid growth of Quantum Computing^1^ has highlighted the relevance of quantum information theory tools within computational sciences.^2–5^ This convergence has promoted a reciprocal exchange of ideas between computational sciences and Quantum Computing, moving us closer to the era of hybrid quantum-classical computing.

In the field of Quantum Chemistry,^6,7^ this exchange has enabled for existing algorithms^8–12^ to benefit from the inclusion of chemical concepts, exemplified by the UCCSD ansatz^13–17^ for variational quantum chemistry algorithms. Additionally, advancements in quantum information theory have benefited classical quantum chemistry by enhancing the understanding of chemical properties,^18–24^ monitor reaction paths^25,26^ and introducing tools that help focusing computational efforts where they are most needed.^27–29^

Despite increasing interest in quantum information theory analysis within computational sciences, most existing works rely on Tensor Network methods, which facilitate the intuitive handling of wave functions to extract quantum information properties. Within this framework, the Density Matrix Renormalization Group (DMRG)^30–34^ algorithm has proven to be a highly capable and versatile tool,^35^ retrieving high-quality wave functions in a description, which can then be analyzed using various techniques to extract quantum information.

However, certain applications of tensor network methods exhibit significant limitations in system size and quality. In quantum chemistry, the nonlocality of the electronic Hamiltonian makes DMRG calculations for systems larger than a few hundred qubits impractical, leaving a gap for quantum information theoretical analysis on larger systems.

This work tries to fill this gap by introducing the Sparse Quantum State Analysis (SparQ) tool. We here propose a method to effectively calculate the density matrix of quantum information theory for any wave function that is sparse in its definition space. The main focus of this procedure is to manipulate quantum chemistry’s wave functions, coming from Post-Hartree–Fock (HF) methods, directly to the qubit space through a Fermion-to-qubit mapping.^36,37^ Furthermore, given the inspiration from which this work arises,^38,39^ it falls directly in line with the mentioned concept of bringing Quantum Chemistry and quantum computing closer.

The manuscript is organized as follows, at the beginning we will dive deeper into the necessity of a tool different from tensor networks to analyze wave functions, justifying it in part with the chemical background that has sparked the idea for this work. Following this track, we will show how to encode Fermionic wave function to the qubit space.

We then proceed with a detailed explanation of the developed tools, with an analysis of the performance and computational costs of the introduced procedures. We conclude by proposing potential usage of the method and by showing practical applications.

## 2 Wave Function Representations and Sparsity in Quantum Chemistry Methods

Given the focus of the authors and the method’s primary application, this section is dedicated to giving a context to the use of wave functions in quantum chemistry and to show how one can map them to the qubit space. However, since this is not essential for understanding the quantum information part of this work, we invite a reader solely interested in analyzing sparse wave functions in qubit space to divert over Section 4 and forward. In quantum chemistry, the MPS-based DMRG^34^ has been so far the method used for quantum information theoretical analysis with few noticeable exceptions posed by^40,41^ making use of an approximation of.^29^ However, the DMRG algorithm cannot handle quantum chemistry applications involving more than a few hundred qubits, which correspond (roughly) to the same amount of spin orbitals. This is due to the scaling of the DMRG algorithm depending heavily on the so-called *bond dimension χ*, with the scaling being lower bounded by *O*(*N* ^4^χ^2^) + *O*(*N* ^3^χ^3^).^42^ This scaling, even if particularly appealing compared to other methods for small to medium sized systems, quickly becomes unmanageable, especially given the growing requirements in terms of χ with growing system sizes.

By contrast, many Post-HF methods can achieve a total scaling limited by HF itself (*O*(*N* ^4^)) depending on the choice of method, with higher scaling methods used to improve precision, with the famous example of *O*(*N* ^5^) for MP2 ^43^ or *O*(*N* ^6^) for CISD/CCSD. This renders possible calculations on up to thousands of spin orbitals (∼qubits). Clearly, accurate algorithms as DMRG should not be so unfairly compared to perturbation theory (or other low-quality) methods, however, the possibility of generalizing quantum information analysis by using wave functions coming from other methods could extend its reach to previously unfeasible systems.

For completeness, we remark that other techniques, based on transition operators,^22,29,44^ also apply to methods different than DMRG, since the necessary ingredients are only some terms of the n-body operators. However, besides the limited application that they find within other Post-HF methods, a transition-operator-based study can only be undergone for density matrices of few qubits, given the complexity of the derivation of these operators.

The stated generalizing purpose raises the point of bringing Fermionic wave functions defined on the Fock space ( F ) to the separable space of the qubits. This conceptually nontrivial task is handled by the next subsection. For what concerns us for the moment, the wave functions under scope can always be written as follows

$$\left|\psi \rangle =\underset{i={\left\{0,1\right\}}^{N}}{\sum }c_{i}|i\right\rangle$$ 1

where each |**i**⟩ is a SD, a vector of a basis of F .

Generally, such an expression implies at most an exponential number of states in the number of Spin Orbitals. This exponential cost cannot be overcome by treating the whole space in a statevector fashion, as one would normally do. Furthermore, while not valid for all existing ones, most Post-HF methods such as MP2,^43^ Configuration Interaction (CI),^7^ and their multireference variants,^45–48^ employ relatively few states out of the exponentially many in eq 1, this is because they usually rely only on a limited number of excitations. For example, in the case of the common double excitations limit, the expansion in the wave function of eq 1 grows as the number of possible double excitations, which scales as *O*(*n*_*o*_^2^ *n*_*v*_^2^), for *n*_*o*_ representing the occupied orbitals and *n*_*v*_ the unoccupied ones. The actual space is then reduced to a polynomial number of SD when the total space is still of exponential dimension, part of this work is dedicated to exploiting this sparsity for efficient treatment of the wave function.

### 2.1 Encoding of Fermionic Wave Functions

One crucial point about the Fock space F is that, due to the antisymmetry of the Fermionic wave functions, the space is not separable, as a consequence of the indistinguishability of quantum particles. Thus, it is described with a direct sum of antisymmetrized products (for Fermions) of single-particle Hilbert space. On the other hand, the quantum states involved in quantum information theory belong to the tensor product single-particle states as a consequence of the distinguishability of particles and the resulting space is separable. By taking into account the Fermionic-to-qubit mappings, the states belonging to the Fock space can be represented in a qubit quantum register by preserving the algebra of the creation and annihilation operators. It follows that, in qubits space, we can perform any operation that would be executable in the Fock space.

Beginning with the wave function definition in eq 1, we demonstrate how to map the corresponding Fermionic excitations to Pauli operators in the separable qubits space using a Fermionic-to-qubit mapping.

We notice that the following notation, just as the work itself, has been strongly influenced by the work of Miller et al.,^37^ to which we refer for further deepening of the theory regarding Majorana operators and Fermionic-to-qubit mappings in general.

To maintain a consistent notation for both Pauli operators, SD and eq 1, **i** will represent the binary string with ones indicating occupied Fermionic modes (Spin Orbitals). This means that, for a system of *N* Fermionic modes (the spin orbitals of the systems we consider)

2 $$a_{i}^{†}=\prod_{j=1}^{N}{\left(a_{j}^{†}\right)}^{i_{j}},i_{j}=0,1,j=1,\cdot \cdot \cdot ,N$$

Consequently, the operator *a*_**i**_^†^ applies the ordered creation operators of the ones in the **i** binary string. In this framework, every state belonging to the Fock space can be represented as

3 $$\left|\psi \rangle =\sum_{i={\left\{0,1\right\}}^{N}}c_{i}a_{i}^{†}|⌀\right\rangle$$

where |⌀⟩ is the vacuum state. As an example, for a system described with 4 spin orbitals, the string **i** = {1010} is associated with the following Slater determinant *a*_{**1010**}_^†^|⌀⟩ = *a*_1_^†^ *a*_3_^†^|⌀⟩ = |1010⟩.

The Fock space generated by *N* Fermionic modes can be mapped to the *N*-partite Hilbert space­(the qubits space) by means of a *Fermionic-to-qubit* mapping, referred to as K . The mapping associates to each creation operator *a*_*j*_^†^ the corresponding operator in the qubits space *a*_*q*,*j*_^†^ for each *j* = 1, ···, *N*. The mapping preserves the Fermi algebra associated with the Fermionic modes

4 $$\begin{array}{l} \left\{a_{i},a_{j}\}=\{a_{q,i},a_{q,j}\right\}\\ \left\{a_{i}^{†},a_{j}^{†}\right\}=\left\{a_{q,i}^{†},a_{q,j}^{†}\right\}\\ \{a_{i},a_{j}^{†}\}=\{a_{q,i},a_{q,j}^{†}\}=\delta_{i,j}1 \end{array}$$

In ref 37 it is shown how there exists a class of Fermionic-to-qubit mappings such that the vacuum state |⌀⟩ is mapped to the vector of the computational basis with all zeros |**0**⟩. Assuming that the used K belongs to this class, we summarize the action of the mapping as follows

5 K(|⌀⟩)=|0⟩

6 $$K\left(a_{j}^{†}\right)=a_{q,j}^{†}=\frac{1}{2}\left(\gamma_{q}^{2j-1}-i\gamma_{q}^{2j}\right)$$

7 $$K\left(a_{j}\right)=a_{q,j}=\frac{1}{2}\left(\gamma_{q}^{2j-1}+i\gamma_{q}^{2j}\right)$$

where γ_*q*_^*l*^ are the Majorana strings, i.e., anticommuting operators in the qubit space associated with the Majorana Fermions defined in the Fock space. Now, given this framework, we have Majorana operators of the following form

8 $$\gamma_{q}^{l}=\overset{N}{\underset{m=1}{⊗}}\sigma_{l,m}$$

where σ_*l*,*m*_ = *X*_*m*_, *Y*_*m*_, *Z*_*m*_, *I*_*m*_ are the Pauli matrices with the identity, acting on qubit *m* = 1, ···, *N*.

To each creation operator we can associate the value of the Hamming distance calculated taking into account the associated Majorana strings

9 $$H\left(a_{q,j}^{†}\right)=H\left(\gamma_{q}^{2j},\gamma_{q}^{2j-1}\right)=\sum_{m=1}^{N}\delta_{\sigma_{2j,m},\sigma_{2j-1,m}}$$

Suppose that for each of the *N* Fermionic modes the Hamming distance is greater than one. In this general case, a product of *P* Fermionic modes is required to store 2^*P*^ Majorana strings to encode the wave function. This is the case of the parity mapping, as can be easily understood by considering the corresponding tree shown in.^37^ However, generally, this prohibitive cost can be reduced if just a subset of the majorana pairs associated with a given excitation operator has Hamming distance greater than 1. More information regarding the procedure for such cases is given in Appendix A.

### 2.2 Jordan Wigner Mapping

The Jordan–Wigner^36^ mapping, one of the most used and ancient mappings, has Hamming distance equal to one for any mode. This fact renders particularly convenient the encoding of a wave function in this mapping. The Jordan–Wigner mapping encodes on each qubit the occupation number of the corresponding Fermionic mode and it is defined as

10 $$\sigma_{l,m}=\{\begin{array}{ll} I_{m} & \mathrm{if}\left[\frac{l}{2}\right]>m\\ X_{m} & \mathrm{if}\left[\frac{l+1}{2}\right]=m\\ Y_{m} & \mathrm{if}\left[\frac{l}{2}\right]=m\\ Z_{m} & \mathrm{if}\left[\frac{l}{2}\right]<m \end{array}$$

Using this definition, eq 7 assumes the following form

11 $$\begin{array}{l} a_{q,j}^{†}=\overset{N}{\underset{m=1}{⊗}}\epsilon_{j,m}\;\mathrm{with}\\ \epsilon_{j,m}=\{\begin{array}{l} \sigma_{2j,m},,\mathrm{if}\;\sigma_{2j,m}=\sigma_{2j+1,m}\\ \frac{1}{2}\left(\sigma_{2j,m}-i\sigma_{2j+1,m}\right),,\mathrm{otherwise} \end{array} \end{array}$$

This allows us to rewrite a single SD of eq 3 as

12 $$K\left(a_{i}^{†}\right)=a_{q,i}^{†}=\overset{N}{\underset{m=1}{⊗}}\prod_{l=1}^{N}{\left(\epsilon_{l,m}\right)}^{i_{l}}$$

We can now rewrite directly eq 3 as

13 |ψ⟩=∑i={0,1}Nciaq,i†|0⟩=∑i={0,1}Nci(⊗m=1N∏l=1N(ϵl,m)il)|0⟩

Now, to pave the way for the usage of wave functions of this form in the following Section 4, we write the wave function as follow

14 $$\left|\psi \right\rangle =\sum_{i={\left\{0,1\right\}}^{N}}c_{i}\left(\overset{N}{\underset{m=1}{⊗}}|\psi \rangle_{i,m}\right)=\sum_{i={\left\{0,1\right\}}^{N}}c_{i}|\psi \rangle_{i}$$

where

15 $$\left|\psi \rangle_{i,m}=\epsilon_{i,m}\right|0\rangle_{m},,\epsilon_{i,m}=\prod_{l=1}^{N}{\left(\epsilon_{l,m}\right)}^{i_{l}}$$

and

16 $${\left|\psi \right\rangle }_{i}=\overset{N}{\underset{m=1}{⊗}}{\left|\psi \right\rangle }_{i,m}$$

Our ket is now written as a sum of tensor products of single-qubit wave functions |ψ⟩_*i*,*m*_. The eq 14 is the final expression for a generic wave function represented on a qubit space, where the sum runs over all the possible SD. However, given the focus of the present work on Post-Hartree–Fock CI-based methods, this sum only runs over the possible excitations on a reference wave function.

One disclaimer, for the simple case of the Jordan Wigner mapping, one could also map the wave function by hand, avoiding this lengthy procedure, as the *a* ^†^ operators for this mapping are quite simple and translate directly eq 1 to a binary expression over the qubit space, so that the following is true

17 $$\sum_{i}c_{i}{\left|\psi \right\rangle }_{i}=\sum_{i}k_{i}|i\rangle$$

where |**i**⟩ is binary ket. On the other hand, this is equivalent to realizing that in eqs 11 and 15, no superposition is created by any ϵ_*l*,*m*_ |0⟩_*m*_, as it can be seen by noting that

18 $$\epsilon_{i,m}{\left|0\right\rangle }_{m}=\{\begin{array}{ll} {\left|0\right\rangle }_{m} & \mathrm{if}\;i_{m}=0\\ \pm {\left|1\right\rangle }_{m} & \mathrm{if}\;i_{m}=1 \end{array}$$

So, for each *m* we never obtain a linear combination of the two states. This will be used in the following to reduce the computational cost of the trace operator.

The long process for mapping the wave function must then be considered for more elaborate mappings, which are built upon the technique explained in this section (Appendix A).

## 3 Quantum Information

The primary objective of this work is to develop an efficient method for computing density matrices traced on subspaces for non-MPS wave functions. To achieve this, we must first evaluate the general complexity of such analyses.

In quantum information theory, analyses typically occur in a Hilbert space H , where each vector represents a wave function $|\psi \rangle_{H}$ . In quantum computing, this Hilbert space consists of multiple separable two-dimensional spaces known as *qubits*. The entire space of *N* qubits H is

19 $$H=\overset{N}{\underset{l}{⊗}}H_{l}$$

with a total dimension of H is 2^*N*^.^49^

Given a wave function |ϕ⟩∈H , its Density Matrix can be defined over the space of *Endomorphisms* of H , End(H) , such that

20 $$\rho_{\phi }=\left|\phi \right\rangle \left\langle \phi \right|$$

This state description captures general quantum or statistical properties, but it requires a quadratic cost in terms of the components of the wave function ϕ. Given the exponential dimension of the space in relation to the number of qubits, analyzing the properties of wave functions and density matrices within this space is impractical.

To reach the goal, there is a need to make use of the sparse property of the wave functions of chemical systems discussed in Section 2.

For a bipartite quantum system with Hilbert spaces $H_{A}$ and $H_{B}$ , the partial trace over subsystem *B* of the density matrix ρ_*AB*_ is defined as

21 ρA=Tr(ρAB)B=∑i⟨i|ρAB|i⟩BB

Where |*i*⟩_*B*_ are any orthonormal basis of the Hilbert space B. The primary use of the traced density matrix is to calculate the Von Neumann (or quantum) mutual information.^50^

**Definition 1** Consider two Hilbert spaces $H_{A}$ and $H_{B}$ , and let $\rho_{\mathrm{AB}}\in H_{A}⊗H_{B}$ be a density matrix. The quantum mutual information *I* of ρ_*AB*_ is given by

22 $$I\left(A,B\right)=\left(S\left(A\right)+S\left(B\right)-S\left(A,B\right)\right)\left(1-\delta_{\mathrm{AB}}\right)$$

where *S* represents the von Neumann entropy defined as

23 $$\begin{array}{l} S\left(A,B\right)=-\mathrm{tr}\left(\rho_{\mathrm{AB}}\log\left(\rho_{\mathrm{AB}}\right)\right)\\ S\left(A\right)=-\mathrm{tr}\left(\rho_{A}\log\left(\rho_{A}\right)\right)\\ \rho_{A}={\mathrm{tr}}_{B}\left(\rho_{\mathrm{AB}}\right) \end{array}$$

Following eq 23, for an N-partite Hilbert space $H_{N}$ and a quantum state expressed by $\rho \in H_{N}$ , we will often refer to the mutual information matrix *I*(*i, j*), obtained tracing out all subsystems except *i*, *j*, i.e., A,B of eq 23. In our use case, the subsystems consist of *N* mapped spin–orbitals. Notably, such a matrix is inherently symmetric, with zero values along the diagonal (by convention, as diagonal values are undefined).

The mutual information defined in eq 22 has been used in the past^24,27,29,35,51,52^ for its flexibility in describing correlation and for its further ability to discern high levels of entanglement.

In the following we will also consider the classical counterpart, the Shannon entropy, which will be useful as a comparison to the von Neumann. Shannon entropy is calculated by evolving the density matrix through a measuring channel as for the following equation

24 $$\begin{array}{l} p\left(i\right)=\left\langle i\right|\rho \left|i\right\rangle \\ M\left(\rho \right)=\sum_{i}p\left(i\right)\left|i\right\rangle \left\langle i\right| \end{array}$$

This channel collapses the wave function rendering it a classical statistical distribution with probabilities *p*(*i*) over the space |*i*⟩, which in our case is the computational basis. This quantity has been recently used to show that orbital correlation is essentially classical when natural orbitals are considered.^53^

As a last tool, we introduce the *Purity* P , which is a quantity used to estimate how close a quantum state is from being pure. It is defined as

25 $$P=\mathrm{tr}\left(\rho^{2}\right)$$

## 4 SparQ

This section describe the procedure to retrieve efficiently quantities such as the trace and mutual information from any sparse wave function in qubit space. Hereafter, since we will no longer refer to the Fock space, we will drop the “*q*” in the equations as we always refer to the qubit space.

For completeness, we pose the attention of the reader to the two ways of representing sparse wave functions.

26 $$\left|\psi \right\rangle =\sum_{j}\lambda_{j}\left(\overset{N}{\underset{m=1}{⊗}}|\psi \rangle_{j,m}\right)$$

The first kind of wave function is the one expressed by eq 26, which is composed of a sum of tensor products of single-qubit wave functions, these wave functions are indexed by *j*, and they sum to whichever number of components the treated wave function has. The second, less general representation is the binary representation ([Disp-formula eq27]), which in previous sections and the following ones has been referred to with the index **i**, and in which each |**i**⟩ is the ket of the relative binary excitation levels of the qubits.

27 $$\left|\psi \right\rangle =\sum_{i={\left\{0,1\right\}}^{N}}k_{i}\left|i\right\rangle$$

In both notations, the main aim is to treat only a limited number of nonzero λ_*j*_/*k*_**i**_. Moreover, we notice that at this point the indexing has only counting purposes, as for eq 26 there cannot be any particular order.

To clarify the differences between the two formalisms, we can take as an example the wave function on two qubits $\left|\phi \right\rangle =\left(\frac{1}{\sqrt{2}}\left(\left|0\rangle_{0}+\right|1\rangle_{0}\right)\right)⊗\left(\frac{1}{\sqrt{2}}\left(\left|0\rangle_{1}+\right|1\rangle_{1}\right)\right)$ . Clearly, by calling ${\left|+\right\rangle }_{0}=\frac{1}{\sqrt{2}}({\left|0\right\rangle }_{0}+|1\rangle_{0})$ we can rewrite |ϕ⟩ = |+⟩_0_ ⊗ |+⟩_1_ following formalism ([Disp-formula eq26]), however, we could just as well expand it in the complete binary form $\left|\phi \right\rangle =\frac{1}{2}\left(\left|00\right\rangle +\left|01\right\rangle +\left|10\right\rangle +\left|11\right\rangle \right)$ , which is the formalism of ([Disp-formula eq27]). While it is the same state, it has a different description in the two notations, and also a different computational cost in its expression, both in time and in memory, as the binary form of |ϕ⟩ is not sparse anymore in its space of definition. In the case of the example |ϕ⟩ one could resort to a local change of computational basis, which can even be done independently for each qubit, however, the division between the two formalisms remains, as this change of basis might not be done independently for each state of eq 26.

Beginning with the first of these two notations, we have shown in Appendix A that any wave function can be encoded as eq 26. For Fermionic wave functions under the Jordan-Wigner mapping, this reduces to eq 14, with λ_*j*_ becoming the *c*_**i**_ coefficients as in eq 14.

### 4.1 Partial Trace and Observables

The primary purpose of the procedures explained in this work is to allow the analysis of properties of a quantum state represented as a vector in a separable Hilbert space, with the main motivation being partial traces, a similar work on this topic exists, but it is not general enough to be applied on quantum chemistry.^54^

We can write the density matrix of the mapped state as

28 ρ=|ψ⟩⟨ψ|

that, by using eq 26, becomes

29 $$\left|\psi \right\rangle \left\langle \psi \right|=\sum_{j,j\prime }\lambda_{j}\lambda_{j\prime }^{*}\left(\overset{N}{\underset{m=1}{⊗}}\left(|\psi \rangle_{j,m}\langle \psi {|}_{j\prime ,m}\right)\right)$$

### 4.2 Partial Trace

We now have the tools required to reduce the dimensionality of the qubit space starting from eq 29. The reduced density matrix corresponding to the qubit *k* can be obtained as follows

30 $$\begin{array}{l} \rho_{k}=\mathrm{tr}{\left(\rho \right)}_{1,\cdot \cdot \cdot k-1,k+1,\cdot \cdot \cdot ,N}=\sum_{j,j^{\prime }}\lambda_{j}\lambda_{j\prime }^{*}\\ tr\left(\overset{N}{\underset{m=1,m\neq k}{⊗}}\left(|\psi \rangle_{j,m}\langle \psi {|}_{j\prime ,m}\right)\right)\cdot |\psi \rangle_{j,k}\langle \psi {|}_{j\prime ,k} \end{array}$$

From this definition, it is clear that the scaling of this operation is quadratic in the number of wave function components χ and linear in the number of qubits being traced *N* – 1. Generally, the scaling will be *O*(χ^2^ *N*)

### 4.3 Observables

Measuring observables (*O*) on the qubit space is a straightforward operation defined by

31 $$O=\sum_{k}o_{k}\overset{N}{\underset{m=1}{⊗}}O_{k,m}$$

Where *O*_*k*,*m*_ is a single qubit operator.

32 ⟨ψ|O|ψ⟩=tr(|ψ⟩O⟨ψ|)=tr(∑j,j′,kλjλj′*ok(⊗m=1N(|ψ⟩j,m⟨ψ|j′,mOk,m)))

We note that this operation has a quadratic cost in the number of states and a linear cost in the number of qubits, as described in eq 30, however, there is now also a multiplicative cost in the number of operators defining *O*.

### 4.4 Direct Tracing

The partial trace equation introduced in eq 30 requires evaluating all possible combinations of two states (∑_*j,j*′_) in the outer product defining the density matrix ([Disp-formula eq28]), significantly limiting the number of states χ of the wave function ([Disp-formula eq26]) that can be considered. Additionally, the traced density matrix produced will have the full dimension of the space, scaling exponentially. This further restricts the maximum dimension of the space that can be processed.

To overcome these limitations, we change the representation for the wave function using the binary representation as in eq 27. This approach reduces the cost of tracing from quadratic scaling in χ to linear scaling, significantly lowering the computational cost. We begin by defining the notation, referring back to eq 27, where each of the |**i**⟩ represents a ket in a binary computational basis of the qubit space. To compute the partial trace, we must first define how the various subspace divisions are represented within this binary notation.

Given a ket |**i**⟩ as in eq 27 and a subset of qubits *A* with Hilbert space $H_{A}$ , we represent the reduction of |**i**⟩ to the space of the qubits in *A* as |**i**⟩_*A*_, indicating the binary collection of the qubit states with the following

33 $${\left(|i\rangle \right)}_{A}=|i\rangle_{A}$$

While working with different subsets of qubits, say *A* and *B*, we can still resort to this notation as long as the two subsets of qubits satisfy *A* ∩ *B* = ⌀. Furthermore, representing with N the set of all qubits, if A∪B=N , then |**i**⟩ = |**i**⟩_*A*_ ⊗ |**i**⟩_*B*_. The direct tracing method is based on this specific case. In order to achieve the best scaling of the partial trace, we make the assumption of starting with the wave function instead of already partially traced density matrices. We then have that *B* can be regarded as *A̅*, to summarize this framework

34 $$\rho_{A}=\mathrm{tr}{\left(\rho_{N}\right)}_{B}=\mathrm{tr}{\left(\rho_{N}\right)}_{\mathrm{A̅}}=\mathrm{tr}(\left|\psi \right\rangle \left\langle \psi \right|{)}_{\mathrm{A̅}}$$

With this notation in mind, we can now retake eq 21 to see how there is only an intrinsic linear cost in the definition, which is brought by the summation over all computational basis states of *A̅*.

Furthermore, since we are working with a sparsely defined vector |ψ⟩ ([Disp-formula eq27]) with χ components, the possible reductions to the space *A* ® are going to be at most min­(2^|*A*^®^|^, χ).

We can now see some differences between this approach and the one in eq 29: Since in the latter the state did not have any intrinsic structure nor order, one could only construct the whole density matrix via outer product of eq 28 and only then work on tracing part of the qubits.

Instead, the aim is now to start with $\left|\psi \right\rangle \in H_{N}$ and obtain the density matrix ρ_*A*_, defined as follow

35 $$\rho_{A}=\mathrm{tr}(\left|\psi \right\rangle \left\langle \psi \right|{)}_{\mathrm{A̅}}$$

To obtain a better scaling, and therefore exploiting the linearity of ([Disp-formula eq21]), the main idea is to consider the density matrix never doing the outer product directly as in eq 30, but rather to only execute the outer product for the combination with the same component in the space *A̅*. In other words, while with eq 30 we first save the density matrix and then operate on it, here we directly use the coefficients of the wave function. Moreover, we note that in eq 30 the resulting traced density matrix could not always be expressed directly as it is in terms of the computational basis in *A*, or else it would take full exponential form. In this notation instead, we will obtain the density matrix directly in a sparse form linked to the computational basis of *A*. This will allow any other kind of sparse operation on the density matrix.

Elucidating the pseudocode scheme reported in Algorithm 1, the procedure consists of the following steps:

1. We start by doing an iteration over all the binary strings in |ψ⟩ ([Disp-formula eq27]) (line 3), during the iterations we save a dictionary γ_*A*_ whose keys are all the existent binary strings in |**i**⟩_*A*_, the items of the dictionary are a set of |**i**⟩*_A_ ®*.
  The dictionary only saves each binary occurrence of *A* once (line 5), by adding another element to the γ_*A*_[|**i**⟩_*A*_] each time there is the same occurrence of a |**i**⟩_*A*_ (line 7). The binary strings in *A*, as keys of the dictionary, will then represent the effective space $H_{A}$ (line 9).
2. After having initialized to zero the density matrix $\rho_{H_{A}}$ to be filled, remembering eq 21, we find the value of coefficient of the density matrix relative to the |**a**⟩ ⟨**a**′|, with $\left|a\right\rangle ,\left|a^{\prime }\right\rangle \in H_{A}$ , by considering all the extensions | **a** ⟩ to the traced space *A* in common between γ_*A*_[|**a**⟩_*A*_] and γ_*A*_[|**a**′⟩_*A*_], i.e.
$$\sum_{|\mathrm{a̅}\rangle \in \gamma_{A}\left[|a\rangle_{A}\right]\cap \gamma_{A}\left[|a^{\prime }\rangle_{A}\right]}\left\langle \mathrm{a̅}\right|\left|i\right\rangle \left\langle j\right|\left|\mathrm{a̅}\right\rangle k_{i}k_{j}^{*}$$
  With |**i**⟩ ← |**a**⟩ ⊗ | **a** ⟩; |**j**⟩ ← |**a**′⟩ ⊗ | **a** ⟩ (line 11–25)

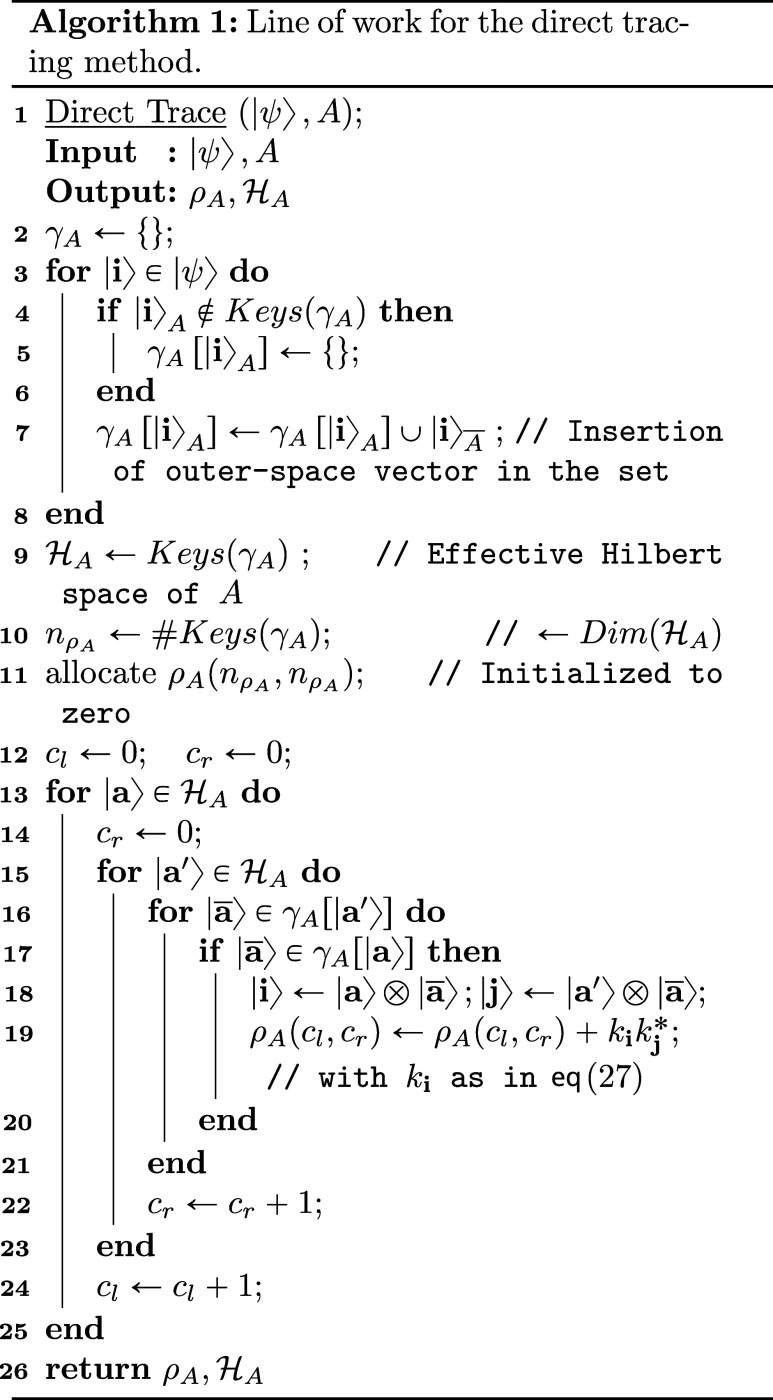


#### 4.4.1 Computational Cost

The difference between the method of eq 30 and 1 can be more clearly understood by considering the computational cost. In the first case, the sparsity of the state is exploited by writing all the elements in a vector in a totally unstructured order, therefore, the use of the density matrix to calculate the partial trace needs to run over all the possible combinations coming out from the execution of the outer product in eq 20. By contrast, the implementation of the direct method 1 saves the data regarding the wave function in grouped sets of dictionaries, which allows for efficient retrieval of only the pieces that contribute to the density matrix with nonzero addition in the partial trace. As shown in with the pseudocode 1, this implementation of the partial trace involves one cycle over all the states in the superposition (line 3) and three for-loops to define precisely ρ_*A*_. However, iterating over all the |**a**′⟩, which are in total *n*_*ρA*_, and | **a** ⟩ entails running over all the existing combinations of |**a**′⟩ ⊗ | **a** ⟩, which are χ. In other words

36 $$\begin{array}{l} \sum_{|a^{\prime }\rangle \in H_{A}}1=n_{\rho_{A}}\\ \sum_{|a^{\prime }\rangle \in H_{A}}\sum_{|\mathrm{a̅}\rangle \in \gamma_{A}\left[|a^{\prime }\rangle \right]}1=\chi \end{array}$$

Therefore, the final time scaling will be *O*(χ*n*_ρ_*A*__). This scaling does not account for the tools needed to identify, save, and retrieve efficiently the correct subspaces at each step, with the most significant being the call to γ_*A*_[|**a**′⟩] and checking the common | **a** ⟩ in lines 16,17 of algorithm 1.

Since these operations necessitates a dictionary, they can be executed within two different frameworks, each requiring a distinct implementation of the dictionary γ_*A*_ and register γ_*A*_[|**a**⟩], We notice however that this only impacts the time requirement for the calls to the dictionary, and thus entails the theory of the partial trace.

1. Using an ordered register, each check, call, or insertion incurs a logarithmic time cost relative to the register size (chapter 13 of ref 55). In a worst-case scenario, there can be χ occurrences in the same register. Since it is reasonable for χ to scale at most polynomially in the number of qubits, checking the register γ_*A*_[|**a**⟩] has a cost scaling as log­(|γ_*A*_[|**a**⟩]|) ∼ log­(*N*).
  The total time scaling would then be *O*(χ*n*_ρ_*A*__log­(*N*))
2. Via Hash tables; each check, call, or insertion is *O*(1) relative to the register size­(chapter 11 of ref 55), although there is an additional cost for hashing the variable **a** ®. This scales linearly in the number of ones in the binary string **a** ®, which is at most N.
  The total time scaling would then at most *O*(χ*n*_ρ_*A*__ × *N*)

In our implementation, we resorted to the second strategy, as by using the particle-hole formalism explained in Appendix C, we only make use of binary strings with a fixed number of ones relative to the number of qubits, rendering the hashing cost a constant and bringing the total cost of the operation back to *O*(χ*n*_ρ_*A*__).

The memory scaling, making such heavy use of hash tables, depends greatly on different implementations, but it can be estimated roughly as a doubling of the weight of the starting wave function |ψ⟩ due to the copying happening in the establishment of the hash table.

This method can be generalized to partial traces of mixed density matrices which cannot be written as ([Disp-formula eq35]), as for example a partially traced density matrix (not of a separable state), but the operation will have again a quadratic scaling in the dimension of the starting ρ.

## 5 Computational Details

In the previous sections, we have introduced two main tools: encoding a sparse Fermionic wave function into qubit space and using these wave functions to retrieve partially traced density matrices.

We now want to present a practical analysis of our SparQ procedure to demonstrate the utility of the method. We use mutual information as the primary metric, defined by eq 22, as it has long been an essential tool for estimating correlations in quantum systems. Its estimation, however, requires the joint density matrix of the two subsystems whose correlation is under investigation.

The simulating framework is characterized by different packages and codes. The SparQ code implementing the trace ([Disp-formula eq21]) has been written in C++ to guarantee easy portability to parallelizing platforms such as OpenMP^58^ while the sparse wave functions are all relative to CISD calculations found with the PySCF^59^ python library. The motivations for choosing CISD over more refined method lye in the CI framework over which SparQ is based, as there is the need of a proper wave function in the CI space. Furthermore, to conduct the analysis of different active space selection methods in 6 we needed a method not including prior choices of active space contrary to most widespread multireference methods at present.

Coming to the actual simulated systems, we used multiple realizations of the hydrogen chain with equispaced hydrogen atoms at a distance of 0.745Å with a 6–31G basis for the analysis of the scaling of the tracing algorithm. In this analysis, the letter *y* will be used to indicate the (varying) number of hydrogen atoms in the chain H_*y*_. To test the encoding in different mappings, we analyzed the water molecule (H_2_O) with an aug-cc-pVTZ^56,57^ basis. At last, to test the full capabilities of the direct tracing algorithm, we studied how the entropy scales for different considered spaces for the benzene (C_6_H_6_) molecule expressed in the cc-pVDZ^56^ basis-set. All the data listed here is also summarized by Table 1. In the following, when the number Slater determinants used for each wave function is stated, it will be meant as the biggest Slater determinants in absolute value of the whole original CISD wave function, with due renormalization procedure.

**Table 1:** Summary of Tested Molecular Systems Together with the Computational Details^a^

Mol.	geometry (Å)	basis-set	#Qubits	tracing method	χ
H_*y*_ chain	H—H distance, 0.745	6–31G	4·*y*	Quadratic Direct	
H_2_O	O 0.0 0.0 0.0	aug-cc-pVTZ^56,57^	184	Direct	1.2·10^5^
	H 0.757 0.586 0.0				
	H – 0.757 0.586 0.0				
C_6_H_6_	C—H distance, 1.085	cc-pVDZ^56^	228	Direct	2.5·10^5^
	C—C distance, 1.390				

a The parameter χ is the number of SD.

The choice of simple systems was motivated by the need for a reliable description of well-understood principles of quantum chemistry. Since this work aims to give quantum information description, what is shown must be compared to known properties of quantum chemistry.

## 6 Results

### 6.1 Scaling Performance

To verify the theoretical scaling and to evaluate the actual computational cost, we use several hydrogen chains H_*y*_ of hydrogen atoms spaced 0.745 Å apart to each other. Although a CISD description of such systems is not appropriate, we have chosen these simple systems only for scaling tests purposes.

The results are summarized in Figure 1, showing the CPU time required to evaluate the partial trace operation from *N* qubits to one qubit. Image (a) shows the time requirements for the quadratic scaling method described by eq 30, while image (b) shows the scaling of the direct tracing method, as explained in Section 4.4.

**Figure 1:**
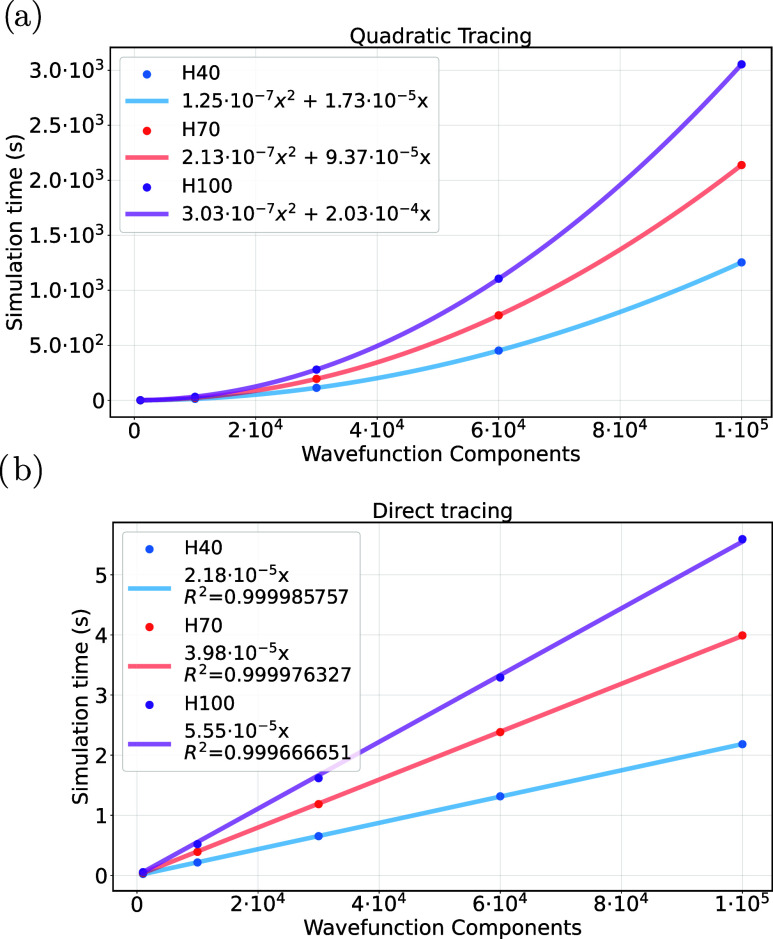
Time requirements for the partial trace from *N* qubits to one qubit over different values of number of components of the wave function (a) Quadratic tracing method of eq 30, (b) Tracing method of pseudocode 1 with linear scaling, with R-squared value for fitting quality estimation. As shown in [Table tbl1], each chain H*y* is expressed with 4*y* qubits.

Looking at image Figure 1-a, we can infer that the scaling of *O*(χ^2^ *N*) for the ([Disp-formula eq30]) is achieved, as by fitting the parabolas not only we found a quadratic scaling for the χ parameter, but we also notice a linear scaling of the fitted coefficient of the quadratic term, coherently with the expected linear growth in *N*. This linear factor is also evident in Figure 1-b, leading to the scaling of *O*(χ × *N*), as evaluated in Section 4.4.1. This is because the results shown in Figure 1 did not make use of the particle-hole notation explained in Appendix C specifically to show the behavior of the used Hash table. Finally, Figure 1-b also presents the R-squared vaue of the fits to quantitatively estimate the linearity of the plots.

Overall, this discussion leads us to believe that both eq 30 and algorithm 1 are valid methods to retrieve the partial trace and mutual information. However, while the quadratically scaling method of eq 30 can hardly handle up to approximately ∼10^5^ wave function components (Figure 1-a), the latter method, maintaining a linear computational cost as shown in Section 4.4.1, can likely be expanded to all the available components of a wave function. Its limitations are primarily due to the resources required for a binary representation of the wave function.

#### 6.1.1 Wave Function in Different Mappings

The first proof of concept involves encoding the Fermionic wave function using different Fermion-to-qubit mappings, as illustrated in Figure 2. We present the mutual information matrices *I*(*i, j*) = *I*_*i,j*_ of the water molecule H_2_O using two different encoding method for the wave function. To retrieve these figures in the parity mapping, we used the particle-hole transformation and CNOTs permutation between mapping, explained respectively in Appendix C and B.

**Figure 2:**
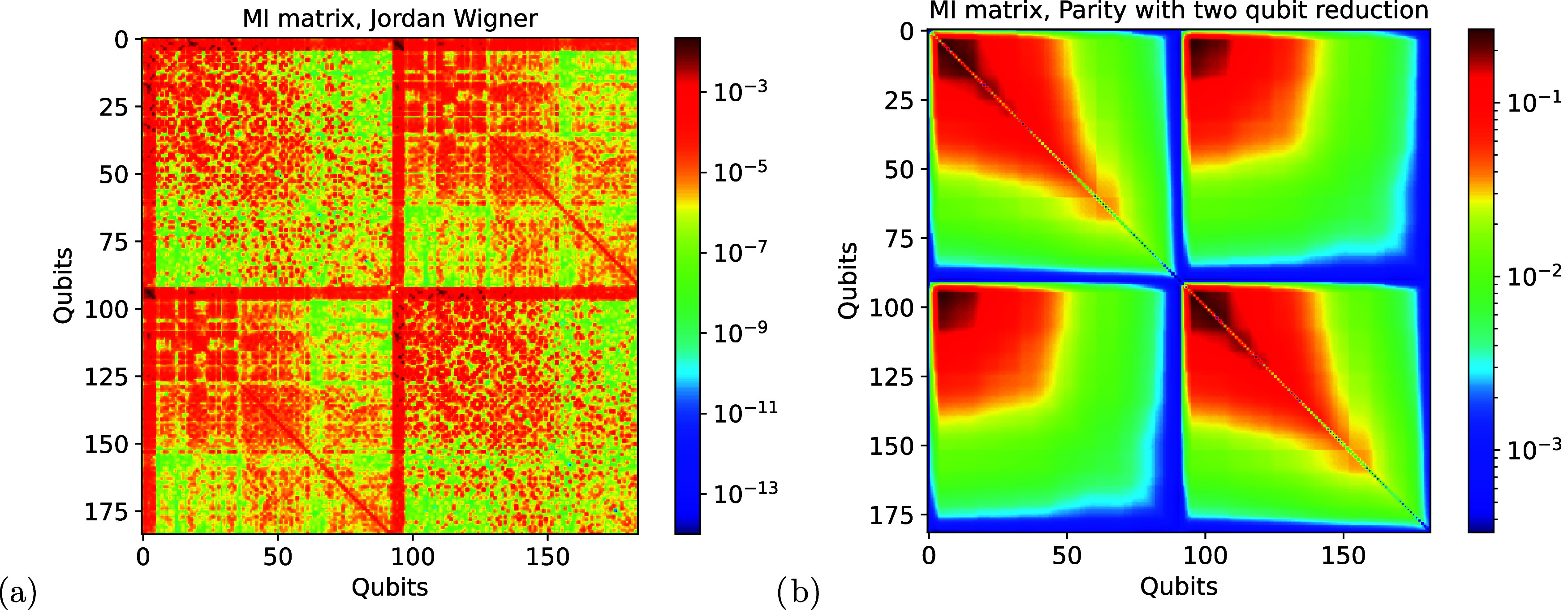
Mutual information for the water molecule at CISD level. Jordan Wigner mapping is reported on the left panel, parity mapping with the two-qubit reduction on the right panel. The starting wave function for the mutual information was the simulation for the water molecule­(H_2_O) as detailed in Table 1. We notice that for both images the apparent subdivision in four quadrants is due to the underlying spin–orbital (up and down) origin of the qubit..

Note that, since each mapping encodes the wave function onto the qubits in a specific way, the resulting mutual information matrices will appear different. The Jordan-Wigner mapping encodes the occupation number of each Fermionic mode, so Figure 2-a shows the correlation between the occupations of the chosen molecular orbitals. In contrast, for the parity mapping, this information is delocalized over the qubits, meaning it cannot be extracted from the corresponding matrix shown in Figure 2-b

### 6.2 Entropy and Active Space Selection

The tool introduced in Section 4.4 has one big advantage compared to the first introduced method of Section 4.2 and to any method based on transition operators,^22,44^ it can retrieve partially traced density matrices defined on an arbitrarily large number of qubits. This is not possible for methods based on transition operator for two main reasons. It first requires expressing each possible |*i*⟩⟨*j*| in terms of multibody terms, and then calculating those terms directly on the wave function and used them in the found expression, both tasks are quite onerous already with two molecular orbital density matrices.^22^ In the present section we would like to bring an analysis only made possible by the introduction of this method, an analysis of the entropies of different descriptions of the benzene molecule. In particular we are interested in comparing different selection methods of active spaces in CAS-SCF.^60^ The choice of active space is not trivial, since some orbitals are more favorable than others in achieving lower energies during the orbital optimization. Several methods have been proposed to help with this task, of which we show the following:

- Fermi level selection.^61^ The active space orbital selection occurs around the Fermi level of the HF occupation of the orbitals. Given its simplicity, it often misses the crucial orbitals if using the most restricted active space possible.
- Natural Orbitals Occupation Number (NOON).^62^ This method analyzes the occupation number of the Natural Orbitals (NO), which are the orbitals diagonalizing the one body reduced density matrix ρ_|ψ⟩,*i,j*_ = ⟨ψ|*a*_*i*_^†^ *a*_*j*_|ψ⟩ of a given Fermionic wave function |ψ⟩.
  The active space orbital selection is performed by including orbitals with occupation numbers that deviate the most from either empty occupation (0 electrons) or full occupation (2 electrons).
  This method has been proven to be quite effective, since the partial occupations are an indication of the presence of correlations in a CI expansion. However, since it relies on some chosen method of obtaining the wave function |ψ⟩ and its related NO, it is not always applicable. Furthermore, one might also want to use different kinds of orbitals for the CAS-SCF, and not the NO.
- AUTO-CAS.^27^ This method is based on an approximate DMRG calculation with low bond dimension. The selection of orbitals is then based on a maximization of the single orbital entropies (see eq 23). This method plays a similar role than the NOON, since an orbital entropy greater than zero also means that the orbital is neither always occupied nor always empty. However, contrary to the NOON selection method, it can be applied to any orbital set.

Our goal is to analyze the behavior of the entropy of active spaces of growing size, investigating differences between the above selecting methods. To do this, we will use the entropy of space *A* as by eq 23 (*S*(ρ_*A*_)) together with the purity of eq 25.

Figure 3-a reports the von Neumann and Shannon entropies as a function of number of orbitals/qubits included in the active space *A*. At the price of redundancy, we stress once more that the entropy shown in Figure 3 is the entropy of the whole space corresponding to the active space.

**Figure 3:**
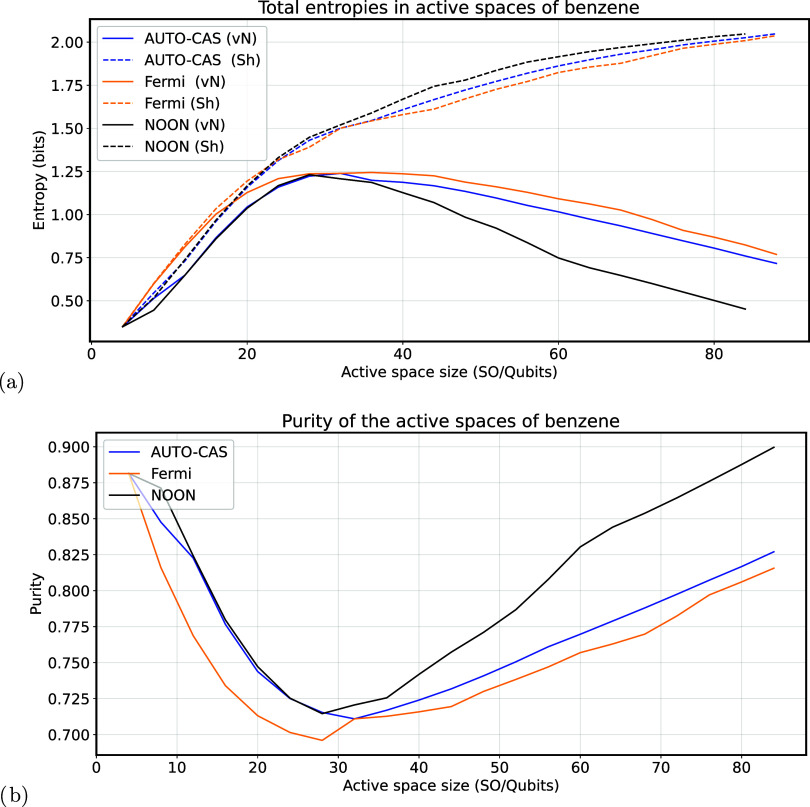
(a) Entropy (*S*(ρ_*A*(*x*)_)­([Disp-formula eq23])) and (b) purity ( $P\left(\rho_{A\left(x\right)}\right)$ ([Disp-formula eq25])) of the density matrix as a function of the active space dimension and selection methods for the benzene molecule. The wave function analyzed is the CISD wave function of benzene with cc-pvdz basis. The Natural Orbitals for the natural orbitals occupation number (NOON) method were retrieved by an iterative procedure (Iterative Natural Orbitals) also based on the CISD wave function. Auto-CAS selects the orbitals maximizing single-orbital entropy, the Fermi method selects orbitals around the Fermi level of the HF orbitals. The continuous line is the von Neumann entropy of the whole space, while the dashed line is the Shannon entropy of the whole space of the state *measured* in the computational basis. Further details can be found in the main text.

The system analyzed is the benzene molecule based on different molecular orbitals sets; HFCO-based (Hartree–Fock Canonical Orbitals) for the Fermi and AUTO-CAS methods, NO for the NOON method, both at the CISD level.

Looking first at the von Neumann entropy, we can see that even if the AUTO-CAS selects the most entropic orbitals, the resulting entropy of the active space is always smaller than the one of the space selected around the Fermi level. While this might seem counterintuitive, it can be explained by considering that the entropy of the untraced density matrix ρ = |ψ⟩ ⟨ψ| must be zero for a pure state. Thus, the AUTO-CAS space is composed of the orbitals that allow to maintain, within the chosen space, the closest description to the original state |ψ⟩ ⟨ψ|. This is proved by Figure 3-b, showing the purity of the density matrices, where the plots show an inverted trend with respect to the entropy one.

The same kind of reasoning can be applied to the general trend of entropy. Looking first at values greater than ∼ 40 qubits, we see a growing trend, motivated by the same concept, namely, that the entropy of the density matrix over the whole space must be 0 and that purity must value to 1.

As for the discussion regarding small-space density matrices, we notice that the local state obtained from the reduction of the HF SD is described by the coefficient of the HF SD itself plus all the contributions which do not concern the selected space. In the same notation as Algorithm 1 _*A*_|*HF*⟩ ⟨*HF*|_*A*_ can be written as

37 |HF⟩⟨HF|AA=cHF2+∑i∈(S,D)∩A̅ci2

Where *A* represents the space selected by the method in Figure 3 and (*S, D*) ∩ *A* represents the space of excitations not involving the space *A*. If we now consider only the diagonal of the density matrix ρ_*A*_ and assume for simplicity that all the other contributions are expressed with a single other state in *A* of weight 1 – _*A*_|*HF*⟩ ⟨*HF*|_*A*_

38 P(ρA)=tr(ρA2)≥tr(M(ρA)2)≃≃A∼0tr((|HF⟩⟨HF|AA001−|HF⟩⟨HF|AA)2)≃|HF⟩⟨HF|AA2

Where we recall that M is as defined in eq 24, the inequality is due to the measurement which can only make a quantum state more mixed and less pure, and can be easily shown by considering the missing pieces in the sum of the found diagonal density matrix. It can now be seen that with space *A* getting smaller, the sum in eq 37 over the complement space will grow bigger, asymptotically to 1.

While the AUTO-CAS data in Figure 3 is based on HFCO, we applied the same method also on the wave function based on NO using natural orbitals based AUTO-CAS, to address the difference between the NOON selection method and AUTO-CAS in these orbitals. This comparison, however, reveals that there is only a small difference, advocating for the similarity of the two methods, at least for simple systems as C_6_H_6_ analyzed in CISD approximation. For this reason, in Figure 3 we only show the NOON between the NO methods, leaving the comparison with NO-based AUTO-CAS for the Supporting Information.

Looking at the NOON data of Figure 3, one can notice an interesting property of the NO, namely, that they allow the greatest purity at a fixed space dimension, evidence for greater effectiveness in describing the system with a restricted space with respect to the HF Canonical orbitals. This extends the already numerous studies regarding the convenient properties of the natural orbitals in quantum information^38,63,64^ and quantum chemistry.^65,66^ One important point being left out from this analysis is the maximum of the von Neumann entropy shown in Figure 3-a around 30 qubits. This particular behavior, while partially justified by the constrained behavior of the entropy at the extrema of Figure 3, still naturally raises the question of whether this maximum has theoretical relevance for an optimal selection of active space. While it seems reasonable to think that the peak of entropy should coincide with maximal efficiency of the CAS-CI description with that space, no proof was found to support this. Furthermore, this discussion could be easily limited by the simplicity of the molecular system under study together with the method for retrieving the wave function.

One last interesting perspective is obtained by looking at the Shannon entropy of the space, which, contrary to the von Neumann one, is a monotone of the space dimension, as marginalization over a probabilistic variable can only decrease classical entropy.

## 7 Conclusions

In this work have introduced SparQ, a method to efficiently calculate partially traced density matrices for sparse wave functions in quantum chemistry. The development of SparQ expands over the limitations of existing methods, mostly based on Density Matrix Renormalization Group (DMRG), which struggles with chemical systems exceeding a few hundred qubits due to the nonlocality of the electronic Hamiltonian. This work therefore also addresses the limitations of studies based on transition operators, by generalizing the trace operator to a wide family of Fermionic-to-qubit mappings and a great number of qubits.

By leveraging the sparsity inherent in many quantum chemistry wave functions, SparQ efficiently encodes Fermionic wave functions into qubit space using Fermion-to-qubit mappings like Jordan-Wigner. This method enables the manipulation and analysis of large-scale quantum systems, significantly extending the reach of quantum information theoretical analysis. The practical utility of SparQ is shown through detailed procedures for the measurement of observables in the qubit space and partial trace of a variable number of qubits scaling linearly in the number of wave function components.

In practical applications, SparQ proves valuable in estimating correlations in quantum systems, as illustrated by the mutual information analysis of the water molecule and the entropy analysis of different possible active spaces of the benzene molecule. Overall, this demonstrated ability to handle large-scale wave functions opens new possibilities for understanding correlations and the behavior of molecular systems.

Future work will focus on refining Fermion-to-qubit mappings, for example, by using the methods illustrated in the Appendices, to further reduce the computational overhead and expand the range of applications for SparQ. The integration of various mapping techniques and the exploration of new application domains will continue to drive the evolution of quantum information analysis in Quantum Chemistry.

In summary, our work may contribute to the ongoing dialogue between quantum computing and quantum chemistry, paving the way for future innovations in hybrid quantum-classical computation and improving the understanding of correlations in quantum chemistry.

## Supplementary Material



## Appendix

### A Mapping Generalization

Even if the main focus of quantum information theory for quantum chemistry has always been within the Jordan-Wigner mapping, one might also be taken in account, for quantum computing reasons,^38,67^ different Fermion-to qubit mapping. We here generalize the mapping procedure for other Fermionic-to-qubit mappings. As we already mentioned in the Majorana operators γ_*q*_^*k*^ must anticommute with each other. Given this anticommuting relation, the Majorana pair must differ in at least one qubit, we can then assert the following

A.1
$$H\left(a_{q,j}^{†}\right)\geq 1$$

where H is the Hamming distance corresponding to the qubit operator *a*_*q*,*j*_^†^ defined above.

Now, while the Jordan–Wigner^36^ mapping, defined as in ([Disp-formula eq10]) can be easily proven to satisfy equality in ([Disp-formula eq39]) for any pair of Majorana operators, general Fermion-to-qubit mappings K do not have this property. In such cases, instead of contracting and summing together the two single-qubit Pauli operators as we done in eq 11, we split each pairs of Majorana strings as follow

A.2
aq,i†|0⟩=∏l=1N(aq,l†)il|0⟩=(aq,1†)i1···(aq,k†)ik···(aq,N†)iN|0⟩=∏l=1N(12γq2l−1−i2γq2l)il|0⟩=∑k=12Ndk(⊗m=1Nσk,m)|0⟩

where **i**_*l*_ = 0, 1 and σ_*k*,*m*_ = *X*_*m*_, *Y*_*m*_, *Z*_*m*_, *I*_*m*_, with *d*_*k*_ coefficients depending on the encoding method and Fermionic modes appearing in **i**. Obviously, the summation runs on a number of elements that depends on the Hamming distance associated with each operator, that are no more than 2^*N*^. For practical cases, this number is drastically reduced. For example, for the Jordan–Wigner encoding method, this expression reduce to eq 12 because the Hamming distance for each operator is equal to one. For a generic wave function

A.3
$$\left|\psi \right\rangle =\sum_{i={\left\{0,1\right\}}^{N}}c_{i}a_{q,i}^{†}\left|0\right\rangle$$

we obtain

A.4
$$\left|\psi \right\rangle =\sum_{i={\left\{0,1\right\}}^{N}}c_{i}\sum_{k=1}^{2^{N}}d_{k}\left(\overset{N}{\underset{m=1}{⊗}}\sigma_{k,m}\right)\left|0\right\rangle$$

that become

A.5
$$\left|\psi \right\rangle =\sum_{j=1}^{4^{N}}\lambda_{j}\left(\overset{N}{\underset{m=1}{⊗}}|\psi \rangle_{j,m}\right)\left|\psi \rangle_{j,m}=\epsilon_{j,m}\right|0\rangle_{m}$$

where ϵ_*j*,*m*_ are single qubit operators and the λ_*j*_ coefficients depends on *c*_**i**_ and *d*_*k*_ ones. We wrote that the summation runs over 4^*N*^ terms because this number is an upper bound but, for practical case, this is no more than polynomial: for example, for the Jordan-Wigner encoding method, we obtain eq 13, thus we have a number of strings equal to the number of Slater determinants in the wave function, with λ_*j*_ being the coefficients of the Slater determinants.

On the mapped wave function |ψ⟩, obtained from the encoding method K , we execute the calculations explained in Section 4. It follow that the cost of these operations is proportional to the cost to encode the wave function, defined as the cost associated with the most expensive Slater determinant belonging to |ψ⟩. So

A.6
$$C_{K}\left(|\psi \rangle \right)=\max_{i\in \left|\psi \right\rangle }2^{P_{i}}$$

where

A.7
$$P_{i}=\sum_{l=1}^{N}i_{l}\Theta \left(H\left(a_{q,l}^{†}\right)-1\right)$$

with

A.8
$$\Theta \left(x\right)=\{\begin{array}{ll} 0 & \mathrm{if}\;x=0\\ 1 & \mathrm{if}\;x>0 \end{array}$$

As can been easily seen, this cost is equal to 1 for the Jordan–Wigner mapping, as has been introduced above.

## Appendix

### B Efficient Mapping Permutation

Given that Fermion-to-qubit mappings are a relatively active field of research,^37,68–71^ we are hopeful that improvement in the understanding of mappings will bring on a further reduction in the cost of encoding wave functions for general mappings, either by reducing $C_{K}$ or by a complete revision of the method. On this line, we can further show how there are already specific techniques to bring down such costs for specific mapping. It is widely recognized that the Parity mapping serves as the dual to the Jordan–Wigner mapping in terms of the information it encodes.

However, it can be shown^72^ that at the level of unitary transformation in the qubit space, the difference between the two can be summarized by a permutation described by the chained application of CNOTs unitary from the first to the last qubit.

B.1
$$\begin{array}{l} U_{\mathrm{JW}\to P}=CNOT_{N-1,N}\cdot \cdot \cdot CNOT_{i-1,i}\cdot \cdot \cdot CNOT_{0,1}\\ a_{P,i}^{†}=U_{\mathrm{JW}\to P}a_{\mathrm{JW},i}^{†}U_{\mathrm{JW}\to P}^{†} \end{array}$$

Considering that each piece in superposition of ([Disp-formula eq13]) for our qubit wave function is generated by *a*_*q*,**i**_^†^, we can rewrite

B.2
aP,i†|0⟩q=UJW→PaJW,i†UJW→P†|0⟩=UJW→PaJW,i†|0⟩=UJW→P(aJW,i†|0⟩)

Since the zero state is an eigenstate of the CNOT and thus *U*_*JW*→*P*_^†^|**0**⟩ = |**0**⟩. At last, since *U*_*JW*→*P*_ (*a*_*JW*,**i**_^†^|**0**⟩) is a classical bit-wise operation, one can conclude that this operation can be efficiently done in any case at no further multiplicative cost from the Jordan Wigner mapping. Finally, we notice that since the only requirement used to establish ([Disp-formula eq48]) was the pure CNOTs composition of *U*_*JW*→*P*_, this procedure can be applied to any mapping in the permutation group of the starting mapping, which in this case is the Jordan Wigner mapping, further details can be found in.^72^

## Appendix

### C Particle–Hole Duality

Up to now we encoded the Fermionic vacuum state into the computational state with all the qubits in the |0⟩ state, so

C.1
|⌀⟩→|0⟩

To reduce the computational effort needed to encode a wave function and perform some operation we take into account the particle-hole transformation. This then brings us to consider the Hartree–Fock state as the vacuum state and to encode it as follow

C.2
|HF⟩→|0⟩

It follow that the operators of the Fermionic modes *a*_*i*_^†^, with *i* = 1, ···, *N*, are transformed as follow

C.3
$$a_{i}^{†}=\{\begin{array}{ll} a_{i}^{†} & \mathrm{if}\;i>n\\ b_{i} & \mathrm{if}\;i\leq n \end{array}$$

where *b*_*i*_ are the annihilation operators for hole particles that are defined for *i* ≤ *n* and *n* = ∑_*k* = 1_^*N*^ ⟨**HF**| *a*_*i*_^†^ *a*_*i*_ |**HF**⟩. Obviously, these operators obey to the Fermi algebra

C.4
$$\begin{array}{l} \left\{b_{i},b_{j}\right\}=\left\{a_{k}^{†},b_{j}^{†}\right\}=\left\{a_{k},b_{j}^{†}\right\}=0\\ \left\{b_{i},b_{j}^{†}\right\}=\delta_{i,j}1 \end{array}$$

with *i*, *j* = 1, ···, *n* and *k* = *n* + 1, ···, *N*. As an example, consider the following state

C.5
$$a_{l}^{†}a_{m}^{†}a_{n}a_{o}\left|\mathrm{HF}\right\rangle =a_{l}^{†}a_{m}^{†}a_{n}a_{o}\prod_{i=1}^{n}a_{i}^{†}\left|⌀\right\rangle$$

To map it on the qubits space, we should apply *n* + 4 operators to the encoded vacuum state |**0**⟩. Taking into account the particle-hole transformation, we obtain that

C.6
$$a_{l}^{†}a_{m}^{†}a_{n}a_{o}\left|\mathrm{HF}\right\rangle \to a_{q,l}^{†}a_{q,m}^{†}a_{q,n}a_{q,o}\left|0\right\rangle$$

so we just apply 4 operators to the vacuum state. The computational cost, in this case, does not depend on the number of particles *n* in the Hartree–Fock state, substantially reducing the overhead.

## Notes

Entropy of different active space sizes with a growing dimension of the space with different selection methods, plot in Natural Orbitals for AUTO-CAS and NOON (PDF)

## Acronyms

CAS: complete active space
CI: configuration interaction
CISD: CI with singles and doubles
DMRG: Density Matrix Renormalization Group
HF: Hartree–Fock
HFCO: Hartree–Fock canonical orbitals
MPS: matrix product state
NO: natural orbitals
NOON: natural orbitals occupation number
SD: slater determinants
SparQ: sparse quantum state analysis
