# Supplementary material for: Quantum Information Theory on Sparse Wave Functions and Applications for Quantum Chemistry
Source: J Phys Chem A. 2025 Sep 9;129(37):8675–88. doi: 10.1021/acs.jpca.5c02137 (PMC12451667; doi:10.1021/acs.jpca.5c02137)
Supplement: Supplementary file 1 [file jp5c02137_si_001.pdf]

Quantum Information Theory on Sparse Wavefunctions and Applications for Quantum Chemistry

Davide Materia[1,2], Leonardo Ratini[3], and Leonardo Guidoni[1]

[1]Dipartimento di Scienze Fisiche e Chimiche, Università degli Studi dell'Aquila, Coppito, L'Aquila, Italy
[2]Dipartimento di Ingegneria e Scienze dell'Informazione e Matematica, Università degli Studi dell'Aquila, Coppito, L'Aquila, Italy
[3]Dipartimento di Scienze Matematiche, Fisiche e Informatiche, Università degli Studi di Parma, Parma, Italy

1 Total entropy and active space, Natural Orbitals

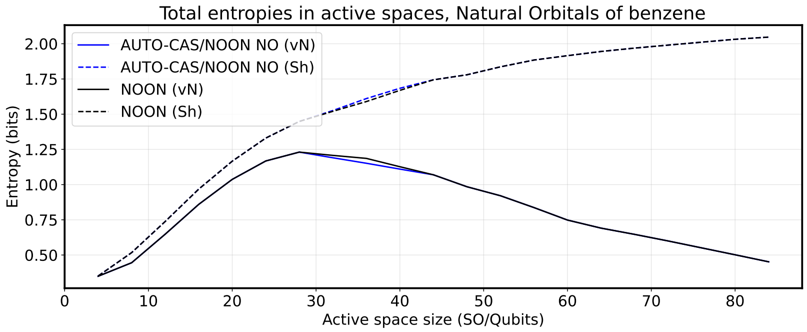

Figure S1: Entropy of the chosen active space with a growing dimension of the space with different selection methods. The wavefunction analyzed is the CISD wavefunction of the C_6H_6 molecule with cc-pvdz basis. The NO for the NOON method were retrieved by an iterative procedure (Iterative Natural Orbitals) also based on the CISD wavefunction. The continuous line is the von Neumann entropy of the whole space, while the dashed line is the Shannon entropy of the whole space of the state *measured* in the computational basis. Further details can be found in the main text.
